# Recommendations for robust and reproducible preclinical research in personalised medicine

**DOI:** 10.1186/s12916-022-02719-0

**Published:** 2023-01-08

**Authors:** Vibeke Fosse, Emanuela Oldoni, Florence Bietrix, Alfredo Budillon, Evangelos P. Daskalopoulos, Maddalena Fratelli, Björn Gerlach, Peter M. A. Groenen, Sabine M. Hölter, Julia M. L. Menon, Ali Mobasheri, Nikki Osborne, Merel Ritskes-Hoitinga, Bettina Ryll, Elmar Schmitt, Anton Ussi, Antonio L. Andreu, Emmet McCormack, on behalf of The PERMIT group, Rita Banzi, Jacques Demotes, Paula Garcia, Chiara Gerardi, Enrico Glaab, Josep Maria Haro, Frank Hulstaert, Lorena San Miguel, Judit Subirana Mirete, Albert Sanchez Niubo, Raphaël Porcher, Armin Rauschenberger, Montserrat Carmona Rodriguez, Cecilia Superchi, Teresa Torres

**Affiliations:** 1 Department of Clinical Science, Centre for Cancer Biomarkers, University of Bergen, Bergen, Norway; 2 EATRIS ERIC, European Infrastructure for Translational Medicine, Amsterdam, The Netherlands; 3 Istituto Nazionale per lo Studio e la Cura dei Tumori “Fondazione G. Pascale” – IRCCS, Naples, Italy; 4 European Commission, Joint Research Centre (JRC), Ispra, Italy; 5 Department of Biochemistry and Molecular Pharmacology, Istituto di Ricerche Farmacologiche Mario Negri IRCCS, Milan, Italy; 6 PAASP GmbH, Guarantors of EQIPD e.V., Central Institute for Mental Health in Mannheim, Mannheim, Germany; 7 Idorsia Pharmaceuticals Ltd., 4123 Allschwil, Switzerland; 8 Helmholtz Munich, Oberschleißheim, Germany; 9 Preclinicaltrials.eu, Netherlands Heart Institute, Utrecht, The Netherlands; 10 Research Unit of Medical Imaging, Physics and Technology, Faculty of Medicine, University of Oulu, 90570 Oulu, Finland; 11 Department of Regenerative Medicine, State Research Institute Centre for Innovative Medicine, LT-08406 Vilnius, Lithuania; 12 Department of Joint Surgery, The First Affiliated Hospital of Sun Yat-sen University, Guangzhou, 510080 China; 13 Departments of Orthopedics, Rheumatology and Clinical Immunology, University Medical Center Utrecht, 508 GA Utrecht, The Netherlands; 14 World Health Organization Collaborating Centre for Public Health Aspects of Musculoskeletal Health and Aging, Université de Liège, B-4000 Liège, Belgium; 15 Responsible Research in Practice, Horsham, UK; 16 Department of Population Health Sciences, IRAS, Faculty of Veterinary Medicine, Utrecht University, Utrecht, The Netherlands; 17 Department of Clinical Medicine, AUGUST, Aarhus University, Aarhus, Denmark; 18 Melanoma Patient Network Europe, Uppsala, Sweden; 19 Global Regulatory Oncology, Merck Healthcare KGaA, Frankfurter Str. 250, 64293 Darmstadt, Germany; 20 Department of Clinical Science, Centre for Pharmacy, The University of Bergen, Bergen, Norway

**Keywords:** Personalised medicine, Translational methods, Preclinical models, Policy, Regulation

## Abstract

**Background:**

Personalised medicine is a medical model that aims to provide tailor-made prevention and treatment strategies for defined groups of individuals. The concept brings new challenges to the translational step, both in clinical relevance and validity of models. We have developed a set of recommendations aimed at improving the robustness of preclinical methods in translational research for personalised medicine.

**Methods:**

These recommendations have been developed following four main steps: (1) a scoping review of the literature with a gap analysis, (2) working sessions with a wide range of experts in the field, (3) a consensus workshop, and (4) preparation of the final set of recommendations.

**Results:**

Despite the progress in developing innovative and complex preclinical model systems, to date there are fundamental deficits in translational methods that prevent the further development of personalised medicine. The literature review highlighted five main gaps, relating to the relevance of experimental models, quality assessment practices, reporting, regulation, and a gap between preclinical and clinical research. We identified five points of focus for the recommendations, based on the consensus reached during the consultation meetings: (1) clinically relevant translational research, (2) robust model development, (3) transparency and education, (4) revised regulation, and (5) interaction with clinical research and patient engagement. Here, we present a set of 15 recommendations aimed at improving the robustness of preclinical methods in translational research for personalised medicine.

**Conclusions:**

Appropriate preclinical models should be an integral contributor to interventional clinical trial success rates, and predictive translational models are a fundamental requirement to realise the dream of personalised medicine. The implementation of these guidelines is ambitious, and it is only through the active involvement of all relevant stakeholders in this field that we will be able to make an impact and effectuate a change which will facilitate improved translation of personalised medicine in the future.

## Background

The “personalised medicine” (PM) paradigm brings promise of delivering tailor-made prevention and treatment strategies for individuals or groups of patients. The idea that individual patients experience disease and response to treatment differently due to variability in genetic and environmental factors is not a new idea, but this recent shift of focus in medicine is driven by advances in multifaceted biological profiling. Improved disease profiling has manifested the need for preclinical models which can generate reliable and predictive data for therapeutic development. The increasing complexity of PM research demands scientific rigour and standardisation of methods in every step [1]. These recommendations were developed in the context of the PERMIT (Personalised Medicine Trials) project [2]. The definition of PM was aligned with the European council conclusion as “a medical model using characterisation of individuals’ phenotypes and genotypes (e.g. molecular profiling, medical imaging, lifestyle data) for tailoring the right therapeutic strategy for the right person at the right time, and/or to determine the predisposition to disease and/or to deliver timely and targeted prevention” [3]. Specifically, the following common operational definition of PM research was applied: a set of comprehensive methods (methodology, statistics, validation, technology) to be applied in the different phases of the development of a personalised approach to treatment, diagnosis, prognosis, or risk prediction. Ideally, robust and reproducible methods should cover all the steps between the generation of the hypothesis (e.g. a given stratum of patients could better respond to a treatment), its validation, and preclinical development, up to the definition of its value in a clinical setting [4–6].

**Fig. 1:**
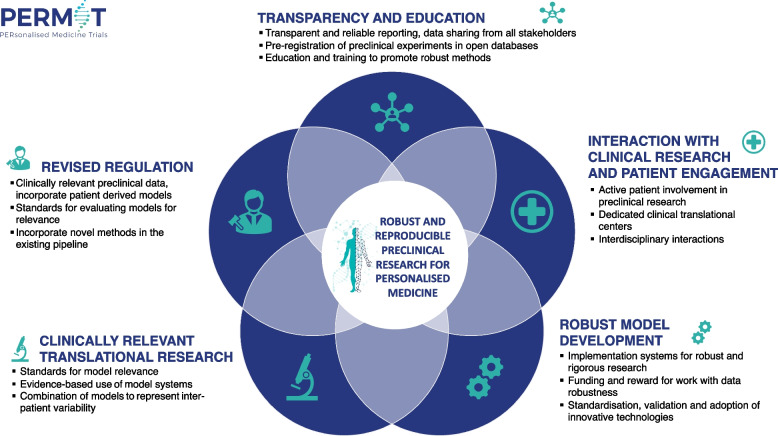
Outline of recommendations for robust translational methods for personalised medicine. The recommendations are focused on five main areas: (1) clinically relevant translational research, (2) Robust model development, (3) transparency and education, (4) revised regulation, and (5) interaction with clinical research, and all these are interconnected

As the first step in building the recommendations, we conducted a comprehensive scoping review of scientific publications and grey literature on the translational steps in PM research programmes and performed a gap analysis [7]. Our results show that, despite the progress in developing innovative and complex preclinical model systems, to date there are fundamental deficits in translational methods, which is one of the obstacles for the implementation of PM. We identified a need for improvements in five critical areas: (1) clinically relevant experimental models; (2) adaptation of standardised protocols, validation procedures, and quality assessment of methods; (3) accurate and transparent reporting; (4) harmonised regulation framework for assessing preclinical evidence; and (5) integration between preclinical and clinical research. These findings are not novel, the issues have been raised by many others before [8–13], but the need for adapted guidelines and standards in preclinical research must be addressed to improve translation and enable PM development. We examined the gaps through a series of consultations with a wide range of experts in preclinical research and identified five points of focus: (1) clinically relevant translational research, (2) robust model development, (3) transparency and education, (4) revised regulation, and (5) interaction with clinical research and patient engagement. These areas are all interconnected, as illustrated in Fig. 1. Here, we introduce recommendations for robust and reproducible preclinical research practices in PM, based on the consensus reached during these consultation meetings.

## Methods

**Fig. 2:**
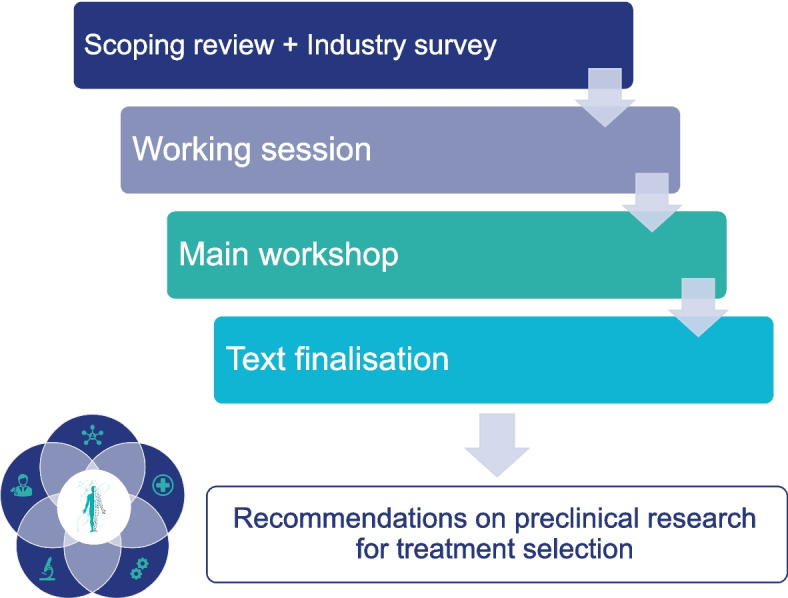
Outline of methodology for developing the recommendations. The methodological approach consisted of four main steps: mapping of current evidence, discussion with field experts, a consensus workshop, and collaborative formulation of the guidelines

These recommendations have been developed following four main steps: (1) scoping review of the literature, (2) working sessions with experts in the field, (3) consensus workshop, and (4) final set of recommendations (Fig. 2).

### Scoping review of the literature

We conducted a scoping review of translational methods for PM to identify relevant gaps, following the Joanna Briggs Institute guidelines. To make the search more manageable, we decided to concentrate on two case models: oncology and brain disorders, which possibly represent the extremes in relation to the availability of preclinical models in PM. The scope was a broad evaluation of the relevance, validity and predictive value of the current preclinical methodologies applied in stratified PM applications. In addition, a survey was sent out to stakeholders working within the pharmaceutical industry, to better understand the approaches that industry follows in developing a patient stratification strategy.

### Working sessions with experts in the field

We hosted four working sessions with PM preclinical experts, between May and June 2021, which aimed to explore different aspects of translational research used for treatment selection. Each session included 8–10 invited experts, who were invited based on their expertise in the field. The four meetings covered the following topics: (1) a working session about preclinical PM approaches within the pharmaceutical industry (*n* = 9 external experts); (2) a working session on in vivo models in PM (*n* = 9 external experts); (3) a working session on in vitro models (organoids, 3D-cell cultures, microphysiological systems) for PM (*n* = 6 external experts); and (4) a working session on in silico models for PM (*n* = 5 external experts).

### Consensus workshop

The consensus workshop was held on September 1, 2021, with experts from the previously organised working sessions and others (*n* = 14 external experts). The focus of this workshop was to discuss the main gaps identified in preclinical methods for PM. The agenda was developed around five main areas: (1) translational research, (2) robust model development, (3) transparency and education, (4) revised regulation, and (5) interaction with clinical research and patient engagement. The aim was to design a framework for the development of recommendations around robust data generation and optimal use of in vivo, in vitro and in silico preclinical models for patient stratification. The consensus approach took the form of an open discussion, not a structured process.

### Final set of recommendations

The authors of this manuscript have formulated the recommendations based on the conclusions of the consensus workshop.

## Results

### Scoping review

A total of 1292 and 1516 records were identified from the oncology and brain disorders search, respectively. Quantitative and qualitative synthesis was performed on a total of 63 oncology and 94 brain disorder studies. In the field of oncology, preclinical models which can recapitulate the patient tumour heterogeneity exist; nevertheless, the approach of modelling patient clustering through this approach is not yet widely used for various reasons. In brain disorders, there is no availability of models which can fully recapitulate patient phenotypes, and little is understood regarding the disease mechanisms occurring at an individual level. The complexity of PM highlights the need for more sophisticated biological systems to assess the integrated mechanisms of response. Emerging models, such as organ-on-chip and in silico models, have been proposed to close the translational gap in the future. However, this relies on technologies which are still in their infancy, and additional fundamental issues in preclinical research remain unsolved. Underlying gaps relating to the relevance of experimental models, quality assessment practices, reporting, regulation, and a gap between preclinical and clinical research must be addressed to achieve a broad implementation of predictive translational models in PM [7].

### Working sessions

The gaps identified in the scoping review were discussed with the field experts during the four separate working sessions. The discussions were topic/model specific; however, the causative explanations and suggestions for improvements were similar across the groups. Detailed reports from each meeting can be found on the open-access platform Zenodo [14].

### Consensus workshop

The common findings from the working sessions were structured into five main categories and presented during the consensus workshop: (1) clinically relevant translational research, (2) robust model development, (3) transparency and education, (4) revised regulation, and (5) interaction with clinical research and patient engagement. The main gaps, potential causes of the gap, and essential points for the recommendations to improve the translational step in personalised medicine are summarised in Table 1. There were presentations from the European Commission on their initiatives for validating and promoting novel non-animal methods, and open science policies. Consensus on the outline of the recommendations was reached through open discussion. The breadth of the points in the discussions has been included in the discussion of each recommendation below. The full report from the workshop is available at the open-access platform Zenodo [14].

**Table 1:** Gap analysis and outline of recommendations for the translational step of personalised medicine

Gap identified	Cause of gap	Recommendation
Lack of clinically relevant experimental models.	Clinically relevant models not emphasised. Complexity of personalised medicine. Need further technological advances.	***Clinically relevant translational research*** Standards for model relevance. Evidence-based use of model systems. Combination of models to represent inter-patient variability.
Lack of standardised protocols, validation procedures, and quality assessment.	Preclinical models often not robust enough for translation. Model validation not academically rewarded, time consuming, and expensive.	***Robust model development*** Implementation systems for robust and rigorous research. Validation, qualification, and adoption of innovative technologies. Funding and reward for work with data robustness.
Lack of accurate reporting, and reporting of negative results, leading to a lack of systematic reviews and meta-analyses on methods.	Non-compliance with reporting recommendations. Academic reward system for publishing positive results. Competitive secrecy from industry.	***Transparency and education*** Transparent and reliable reporting, data sharing from all stakeholders. Preregistration of preclinical experiments in open databases. Education and training to promote robust methods.
Regulation—lack of standards for relevance and robustness of preclinical evidence.	Regulators and ethical committees lack harmonised guidelines on preclinical assessment.	***Revised regulation*** Patient centric, clinically relevant preclinical data. Standards for evaluating models for relevance. Incorporate novel methods in the existing pipeline.
Lack of involvement between preclinical and clinical research.	Mindset of the medical and scientific communities. Need for better definition of patient engagement.	***Interaction with clinical research and patient engagement*** Active patient involvement in preclinical research. Dedicated clinical translational centres. Interdisciplinary interactions.

A subset of the participants from the workshop volunteered as co-authors, and the formulation of the specific recommendations was developed through collaborative writing. The main categories and outline of the recommendations presented in the consensus workshop were only refined, not changed. The recommendations and the stakeholders they address are summarised in Table 2 and elaborated and discussed under each separate topic below.

**Table 2:** Summary of specific recommendations for robust and reproducible preclinical research in personalised medicine and identification of the stakeholder(s) they address

Number	Recommendation	Stakeholder addressed
1	It is imperative that preclinical translational models are assessed and developed to ensure they capture clinically relevant aspects of the disease and are aimed towards the prediction of treatment outcome or prevention	Researchers
2	The selection of preclinical models must be evidence-based, and researchers should demonstrate awareness of the limitations of the model(s) when interpreting results.	Researchers
3	Several models must be used when modelling complex disease, to represent different features of the disease.	Researchers
4	There should be a common implementation framework for robust and rigorous research, to provide reliable preclinical data prior to clinical trials.	Researchers Funders
5	Public funders must support and promote robust model development through specific funding and policies.	Funders
6	Further efforts should be made to validate, qualify, and adopt innovative technologies.	Funders
7	Transparent and reliable reporting and data sharing must be a requirement for both the academic and commercial sectors to improve the quality, credibility, and responsiveness of research	Researchers
8	Pre-registration of preclinical study protocols in open-access databases should be required by research funding bodies and/or research organisations.	Researchers
9	All stakeholders must ensure that the education and training of researchers promote methods for high-quality and reproducible preclinical research.	All
10	Regulators should ensure that preclinical evidence is clinically relevant and encourage incorporation of patient-derived models.	Regulators
11	Regulators and ethics committees reviewing and approving clinical trials should have harmonised guidelines and standards for evaluating preclinical evidence.	Regulators
12	Regulators should facilitate the incorporation of novel patient-derived methods in the drug development pipeline.	Regulators
13	Active patient involvement in PM preclinical research should be facilitated and incentivised through public funders.	Researchers and funders
14	The development and infrastructure of dedicated patient-focused interdisciplinary translational centres should be supported by targeted public funding.	Funders
15	All relevant stakeholders in translational PM development should encourage and facilitate interdisciplinary interactions to address the causes of translational failure and enhance efforts to develop robust research models	All

## Discussion

### Clinically relevant translational research

Despite recent developments of sophisticated and novel methods in preclinical research, there is still a deficiency of models that can reliably replicate patient groups sufficiently to enable benefits from PM to be realised. Only a small proportion of preclinical research performed prior to clinical trials translates into clinical benefit in humans [15], for instance in Alzheimer’s disease the failure rate is 99% [16], and in oncology only 5% of anticancer agents reach the clinic [17]. The complexity of personalised approaches in most diseases makes preclinical model development challenging, perhaps except for those attributed to a simple genetic mutation. In oncology, the field has progressed towards personalising preclinical models through patient-derived xenografts (PDXs) and patient-derived 3D cellular models and organoids. However, despite these complex models being more biologically relevant, they are extremely costly, and there are intrinsic challenges in reproducibility [18, 19].

The interest of the regulatory agencies for innovative and emerging technologies is growing both in Europe [20] and across the Atlantic [21]. Microphysiological systems, such as organ-on-chip models, are promising and could represent a fit-for-purpose personalised aspect of patient disease in the future [22]. They mimic 3D structures and biophysical features of tissues [19], and they are estimated to substantially decrease the costs for the research and development of therapies [23]. Nevertheless, these novel models still need further technological advances, validation, and standardisation in order to be accepted for regulatory purposes [24]. In addition, in this digital era, in silico methods [25], and the use of machine learning and big data [26, 27] are expected to revolutionise PM; however also there, standardisation is a huge issue. Efforts are being made to overcome it, e.g. through projects like EU-STANDS4PM [28], which are in the process of developing an ISO document (ISO/AWI TS 9491-1) on translational standards for these models. The PERMIT project has also addressed this issue through a scoping review and recommendations [6, 29]. The success of such efforts is also dependent upon the development of a global translational medicine community to coordinate interdisciplinary research that can better address unmet medical needs. This is the aim of the Eureka Institute for Translational Medicine [30]. In reality, the currently applied preclinical methods are not always clinically relevant, and their limitations are often overlooked, resulting in a tendency for the over-extrapolation of results [11, 31].

*Recommendation #1: It is imperative that preclinical translational models are assessed and developed to ensure they capture clinically relevant aspects of the disease and are aimed towards the prediction of treatment outcome or prevention.*

There is a lack of harmonised standards to evaluate the advantages and limitations of model systems, and there is currently no formal requirement to assess the clinical relevance of preclinical research. Tools to assess clinical relevance have been described [32–34], intended for use by researchers considering the translational value of preclinical findings to first-in-human clinical trials, the funders of such studies, and regulatory agencies that approve first-in-human studies. The use of systematic reviews for evidence-based decision-making in preclinical research has been advocated for many years [12, 35, 36]. Indeed, there is a growing community of individuals and organisations conducting preclinical systematic reviews and developing tools for researchers [37, 38]. This is vital to have a realistic evaluation of the capabilities and limitations of a model, to avoid a narrow focus on commonly used models, current academic trends, and hype.

*Recommendation #2: The selection of preclinical models must be evidence-based, and researchers should demonstrate awareness of the limitations of the model(s) when interpreting results.*

The complexity of PM and the knowledge gaps in biological processes means that, to date, it is an unrealistic expectation to be able to accurately reflect patient heterogeneity in one model. For instance, modelling the inter-patient variability of the immune system is a key challenge. Deep molecular phenotyping to uncover the heterogeneity of diseases, as well as the variability in response and tolerability of treatments, is crucial for model improvement. A combination of different models, that together represent patient variation, is a more realistic strategy, but requires cross-disciplinary collaborations. To date, the lack of predictive preclinical models reflecting patient heterogeneity means that personalised approaches are mainly developed in the clinical space. An important aspect of preclinical modelling is to provide basic safety data before clinical trials. Inappropriate preclinical models could potentially have severe implications for patient safety, if the model does not represent the exposure-response relationship, of which there are some recent examples in immuno-oncology [39, 40].

*Recommendation #3: Several models must be used when modelling complex disease, to represent different features of the disease.*

The key aspect of preclinical research is to increase the odds that a novel therapeutic mechanism of action will benefit patients, and predictive translational models are a fundamental requirement to realise the dream of PM. This will require more structured interdisciplinary collaborations among all stakeholders, including the patients themselves.

### Robust model development

Rigour in research is paramount for ensuring robust preclinical models and methods. Indeed, the low success rate in the translation of novel therapies to the clinic can also be partly attributed to the fact that there is a lack of internal validity [41–44]. In addition to the clinical relevance tools mentioned above (see Recommendation 1), two recent public-private initiatives have developed approaches for improving quality in preclinical research. For example, Knopp and colleagues present six key principles for experimental design and conduct for preclinical pain studies: (1) be aware of stressors on animals, (2) perform sample size calculations, (3) specify inclusion/exclusion criteria, (4) perform randomisation, (5) allocation concealment, and (6) blinding [45]. Another approach for comprehensively improving internal validity is the recently established EQIPD Quality System [46]. This systematic approach provides guidance on improving experimental design, increasing research data transparency within the lab and implementation of feedback loops. However, there is currently a lack of policies to ensure implementation of such quality processes for a sustainable change. If an assessment of rigour is a requirement for funding, it will provide motivation to train and mentor researchers to implement best practice. In a survey about reproducibility, about 80% of researchers thought that funders and publishers should do more to improve reproducibility [47]. The international funders forum “Ensuring value in Research” [48] has an ongoing initiative about evaluating the quality and translatability of preclinical studies.

*Recommendation #4: There should be a common implementation framework for robust and rigorous research, to provide reliable preclinical data prior to clinical trials.*

Multi-centre studies are a requirement in clinical research to increase the robustness of research data. Such systematic validation and large inter-laboratory studies are desirable for preclinical research as well, and it has been proposed to introduce a “preclinical trial” requirement, where novel therapeutic findings undergo rigorous and independently performed preclinical studies to confirm the robustness of exploratory research findings, before advancing to clinical trials [49].

This would be essential to achieve standardisation and systematic heterogenisation of models, since there is a wide range of biochemical and biomechanical factors which could influence results [50, 51]. Only both standardisation and systematic heterogenisation of methods can improve quality, reduce bias, and improve translation [52, 53]. Such preclinical multi-centre trials are currently explored in at least two funding schemes, the Brazilian Reproducibility network [38], as well as by a funding scheme from the Federal Ministry of Education and Research in Germany [54]. Such preclinical confirmatory funding schemes could improve clinical translation and be models for other organisations. Another obstacle is that the academic system does not routinely reward work related to developing and validating robust research models [3]. Thus, there is a need for targeted funding to cover the costs of validation processes and to recognise the benefits of supporting robust model development.

*Recommendation #5: Public funders must support and promote robust model development through specific funding and policies.*

Research and innovation should be aligned with the needs of society, and quality assurance standards should come from national and European legislators. The gap between academic and pharmaceutical sectors in relation to the rigour of study design, what constitutes a significant effect size, and selective reporting practices, need to be addressed. Recently, there have been some good examples of such efforts for public-private interactions. There is the Innovative Health Initiative (IHI) funding scheme (formerly Innovative Medicine Initiative, IMI) that provides a funding mechanism for consortia with participants from academic institutions, the pharmaceutical industry, and small to midsize entities. The collaboration between these different stakeholders can be seen as successful and many examples are published [55, 56]. Another call, and a potential blueprint for other funders, from the Federal Ministry of Education and Research in Germany, funded 11 academic early drug development projects with the aim to validate the target for potential clinical investigations [57, 58]. Each of these projects needed to have an experienced mentor with a background in industrial research to assure that input with respect to preclinical development is provided. Similarly structured collaboration between academic and commercial sectors should be further facilitated, to address the causes of translational failure and enhance efforts to develop robust research models. The improvement in the culture and practice of research should be viewed as a process of continuous communication and adaptation, not a singular endpoint, according to experience from the QUEST Center for Responsible Research [59].

*Recommendation #6: Further efforts should be made to validate, qualify, and adopt innovative technologies.*

Innovative technologies and emerging approaches based on them, such as organ-on-chip, and in silico models (using machine learning and/or artificial intelligence (AI) on big data), are picking up pace and could transform the way we conduct biomedical research for drug and biomarker development towards PM. Thus, there is a clear need to invest more resources and efforts to drive the adaptation and use of these cutting-edge tools, both to accelerate innovation in human-relevant research and to develop reliable and predictive alternatives to conventional animal models. Developing standards to characterise new models and methods in support of their qualification of specific context of use will be an important step in establishing scientific credibility and building confidence in new technologies for preclinical PM within the regulatory science community. In addition, if the results from standardised models were made public, it would allow comparison across compounds, which could facilitate a faster access to personalised therapies for patients. The recent emergency approval of the mRNA COVID-19 vaccines in 10 months (instead of 10 years) has demonstrated the potential of applying innovative technologies leading to effective vaccines fast; this can form the basis for continuing on this road [60].

### Transparency and education

Transparency in reporting is essential, and if methods and data are not shared in an unbiased and open format, it contributes to the so-called reproducibility crisis [9, 47, 61]. This can occur as a result of many commonly found poor research practices, for example selective reporting of research outcomes or study results, the over-extrapolation of findings, underpowered studies, and more [62]. Reporting in an accurate manner is vital to maximise the quality and reliability of research. Despite calls for transparent reporting from the scientific community [63], the accuracy and quality of reporting have not improved [64, 65]. Initiatives aimed at scientific journals include the MDAR (Materials, Design, Analysis, Reporting) framework to improve research practices through transparent reporting [66]. Many scientific journals also endorse reporting guidelines such as the ARRIVE guidelines for animal experiments [67], but a randomised controlled trial did not find improved compliance from researchers who received a specific editorial request to fill in the ARRIVE checklist, compared to the manuscripts who did not get the specific request [68]. This may, in part, reflect the fact that reporting in adherence with ARRIVE and other guidelines requires researchers to have planned for this when designing their experiments. The PREPARE Guidelines fulfil this purpose for the planning of preclinical studies involving animals [69]. This alone may not be sufficient, suggesting that additional approaches are required to improve reporting that extend beyond the personal conduct of individual researchers [70]. Indeed, experiences from one author (B.G.) working as a quality manager and auditor in different environments suggest that transparency already needs to be fostered at the level of researchers when performing experiments and not only when publishing. In that regard, it seems to be vital that appropriate education in data integrity for young researchers is introduced and labs have a systematic documentation procedure to ensure transparency (see also Recommendation 9).

*Recommendation #7: Transparent and reliable reporting and data sharing must be a requirement for both the academic and commercial sectors to improve the quality, credibility, and responsiveness of research.*

There is a need to improve the ways in which the output of scientific research is evaluated by funding agencies, academic institutions, and other parties, beyond the Journal Impact Factor system. The academic reward system has traditionally been closely linked with journal metrics. The San Francisco Declaration on Research Assessment (DORA) [71] is a set of recommendations to improve the evaluation of research outputs, and it has been endorsed by many universities and organisations. The Leiden manifesto proposes ten principles for improving metrics evaluations [72]. The European Open Science programme is a step in this direction, and open science is now a policy priority for many funders [73–75]. This policy requires recipients of the research and innovation funding grants to make publications available open access, and data accessible in accordance with the FAIR principles (Findable, Accessible, Interoperable and Reusable) [76]. Open science must include all research sectors, including the pharmaceutical industry. Through the principle of making data *as open as possible and as closed as necessary*, it is possible to report methods and share data without compromising competitive interests. Studies have found several factors, both on an individual and institutional level, that can impact the content and effectiveness of open science policies, and which should be taken into consideration when designing such policies [77–79]. The Research Data Alliance [80] have developed an assessment tool based on FAIR criteria compliance [81]. The final aim is to create a transparent and collaborative environment where the public interest is protected, and research results are reliable and robust. Furthermore, building trust on methods and scientific data is highly relevant for improving the robustness and reproducibility of preclinical research. EURL ECVAM of the European Commission’s Joint Research Centre (JRC) co-organised very recently a workshop with several relevant stakeholders in the life sciences publishing sector. The workshop addressed the need to improve the way protocols and methods are described/reported in scientific publications (guaranteeing reproducibility, transferability, transparency, etc.) and a list of actions—which will become publicly available—is currently under development.

There is evidence of publication bias towards novel, positive, or confirmatory results that support the hypothesis being investigated [82, 83]. This focus means that a large amount of preclinical research generating negative, null, or inconclusive results is never disseminated to the scientific community [84]. Researchers who plan, design, conduct, and analyse their studies in accordance with best practice should have equal confidence in the accuracy of all results, irrespective of the outcome. Indeed, good practice includes the definition of inclusion/exclusion criteria in advance of the study so that if there is a scientifically valid reason for not including results in an analysis, then this can be transparently reported. Prospective registration of animal study protocols—as is already common practice in the clinical arena—can also increase the sharing of data and reporting of results [85]. If all animal studies were to be preregistered, this would result in comprehensive animal study protocol databases that researchers could use to help them answer research questions and design new studies, and it would also contribute to improve meta-research and reduce unnecessary duplications [86]. The Netherlands Organisation for Health Research and Development (ZonMW) has started a pilot for mandatory preregistration of animal research, to create transparency of conducted animal studies and enable researchers to learn from each other’s experimental set-up to reduce unnecessary animal use [87]. This pilot can serve as good practice for other funding bodies. There are currently two registries dedicated to preregistration of animal studies, PreclinicalTrials.eu [88] and animalstudyregistry.org [89]; for in vitro and in silico studies there is currently no dedicated platform, but researchers can use open science platforms [90, 91], and preregistration of mathematical models is advocated [92].

*Recommendation #8: Preregistration of preclinical study protocols in open-access databases should be required by research funding bodies and/or research organisations.*

The challenges relating to transparency and reproducibility will need to be addressed to accelerate robust preclinical development for PM. This will require a cultural change across the scientific community. However, it is important to sensitise to the fact that open science can have different implications, both in contribution and use, depending on geographical location, and be significantly different in low-resource research environments [93, 94]. The education and training of young scientists are fundamental to this, and a framework for developing and sharing educational resources has been suggested as a path to improving rigour during the design, conduct, analysis, and reporting of biomedical research [95].

*Recommendation #9: All stakeholders must ensure that the education and training of researchers promote methods for high-quality and reproducible preclinical research.*

To facilitate and effect change in the scientific community, and evoke public engagement, publicly available materials, educational platforms, and initiatives should be developed and promoted. Several initiatives exist already [96, 97], but a systematic strategy is needed to make a real impact.

### Revised regulation

Compared to clinical research, which is strictly controlled, translational science is relatively unrestricted. Preclinical studies must adhere to regulations for good laboratory practice [98, 99], and in addition, animal experiments are regulated by law for the protection of animals used for scientific purposes, e.g. Directive 2010/63/EU in Europe [100]. This legislation, and its equivalents elsewhere in the world, is critical to ensure that the 3Rs principles of humane experimental technique (replace, reduce, refine) are followed. It does, however, reflect minimum standards, not best practice, and does not specifically require the relevance and translational value of animal models to be assessed.

*Recommendation #10: Regulators should ensure that preclinical evidence is clinically relevant and encourage incorporation of patient-derived models.*

Regulators and ethics committees assessing and approving clinical trials commonly lack guidelines and standards, and also often relevant preclinical expertise, for evaluating evidence from preclinical studies. Proposals for assessing preclinical efficacy studies in a structured process have been made [101], but there is no harmonised evaluation methodology yet, resulting in most evidence being assessed on a case-by-case basis.

*Recommendation #11: Regulators and ethics committees reviewing and approving clinical trials should have harmonised guidelines and standards for evaluating preclinical evidence.*

There is a growing number of preclinical patient-derived disease models available. However, an effective and updated regulatory and legislative landscape is required to facilitate the development, validation, and acceptance of new preclinical methodologies in the PM space [102]. A recent case study shows that EMA shortened its timeline for COVID-19 vaccine approval, by reducing the number of requested animal studies and promoting alternative methods [103].

*Recommendation #12: Regulators should facilitate the incorporation of novel patient-derived methods in the drug development pipeline.*

Running parallel regulatory programmes has been proposed to increase confidence in new approaches and to enhance the transition to implement novel methods. This could facilitate a more human-centric approach for translational sciences, by using human cell systems with varying degrees of complexity and combining them with in silico and in vivo studies to define PK parameters and potential toxic (side)effects [104]. Multi-organ body-on-chips have already been developed to simulate whole body (patho)physiology and also account for the absorption, distribution, metabolism, and excretion (ADME) of pharmacological compounds [105]. However, it is important to note that the existing alternative methods are not yet able to simulate complex behaviours or the entire physiology of an intact living organism.

### Interaction with clinical research and patient engagement

Translational research encompasses the activities that link discoveries in the laboratory to the initiation of human clinical trials [106]. It is vital not to lose sight of the human focus in translational endeavours and to involve patients in preclinical research activities, including the definition of research questions that are considered relevant by the patients themselves. To facilitate this, better understanding of the benefits of patient engagement and awareness of methodologies and approaches in preclinical research is needed. Often, insufficient resources, such as time and budget restrictions, are a threat to the inclusion of patients in preclinical studies [107].

*Recommendation #13: Active patient involvement in PM preclinical research should be facilitated and incentivised through public funders.*

This will require a change in the mindset of the scientific community. There is a need for targeted funding for validating robust preclinical models, facilitating stakeholders’ interactions and to create a reward system for human resources for making advances that are sustainable and robust over time.

*Recommendation #14: The development and infrastructure of dedicated patient-focused interdisciplinary translational centres should be supported by targeted public funding.*

Translational research is complex, and it is most beneficial when undertaken as a multi-sector endeavour. The creation and promotion of multidisciplinary groups are key to the aim of improving translational research activities. To achieve this, there must be alignment in the confidence among the relevant stakeholders (researchers, clinicians, patients) of the value of preclinical data [108, 109]. There are reports of failed attempts at introducing personalised approaches, attributed to the lack of consensus among the researchers and primary clinicians [110]. The European Commission recently published a report called Bridging Across Methods in the Biosciences (BeAMS), where working towards a common “language” and making use of insights from social and philosophical studies of science are identified as priorities towards achieving crossdisciplinarity across methods, disciplines, and sectors in biosciences [111]. Thus, it is proposed that the creation of dedicated translational research centres, with knowledge of reliable translational models and the capability to connect it with patients in the clinic, can bridge the preclinical research gaps.

*Recommendation #15: All relevant stakeholders in translational PM development should encourage and facilitate interdisciplinary interactions to address the causes of translational failure and enhance efforts to develop robust research models.*

Collaboration and openness should be in place in a translational setting, bringing together researchers, industry, clinicians, and patients. The creation of a pathway from basic academic research to clinically approved new therapies will probably also require visions for new models of structured collaborations for commercialisation [112].

## Conclusions

The development and validation of robust and predictive preclinical models that can capture clinical phenotypes and enable patient stratification for complex diseases is challenging, but fundamental for further development of personalised approaches. We have presented a set of recommendations aimed at improving the robustness of translational research for PM. These recommendations embrace the whole pipeline of developing individualised therapies, and we encourage an increased patient focus and more interdisciplinary collaboration in every step. The implementation of these guidelines is ambitious, and it is only through the active involvement of all relevant stakeholders in this field that we will be able to make an impact and effectuate a change which will facilitate improved translation of PM in the future.

## Data Availability

Copies of searches and data extraction sheets from the scoping review are publicly available on the online platform Zenodo (https://zenodo.org/record/6087847), as part of the database collection for all scoping reviews conducted in the PERMIT project. The detailed reports from the working sessions and main workshop are also available on Zenodo (https://zenodo.org/deposit/7086703).

## Abbreviations

ARRIVE: Animal Research: Reporting of In Vivo Experiments
DORA: Declaration on Research Assessment
PDXs: Patient-derived xenografts
PERMIT: Personalised Medicine Trials (https://permit-eu.org)
PM: Personalised medicine
3D: Three dimensional
